# FIP200 Methylation by SETD2 Prevents Trim21-Induced Degradation and Preserves Autophagy Initiation

**DOI:** 10.3390/cells11213333

**Published:** 2022-10-22

**Authors:** Yuan Dai, Weijia Luo, Wenjiao Li, Zhishi Chen, Xinjie Wang, Jiang Chang

**Affiliations:** 1 Center for Genomic and Precision Medicine, Institute of Biosciences and Technology, Texas A&M University, Houston, TX 77030, USA; 2 Department of Genetics and Genomic Sciences, Icahn School of Medicine at Mount Sinai, New York, NY 10029, USA

**Keywords:** autophagy, post-translational modification

## Abstract

FIP200, also known as RB1CC1, is a protein that assembles the autophagy initiation complex. Its post-translational modifications and degradation mechanisms are unclear. Upon autophagy activation, we find that FIP200 is methylated at lysine1133 (K1133) by methyltransferase SETD2. We identify the E3 ligase Trim21 to be responsible for FIP200 ubiquitination by targeting K1133, resulting in FIP200 degradation through the ubiquitin–proteasome system. SETD2-induced methylation blocks Trim21-mediated ubiquitination and degradation, preserving autophagy activity. SETD2 and Trim21 orchestrate FIP200 protein stability to achieve dynamic and precise control of autophagy flux.

## 1. Introduction

Macroautophagy (hereafter autophagy) is an evolutionarily conserved mechanism of lysosome-dependent degradation of intracellular components, from proteins to organelles, that helps cells survive stressful conditions [1,2]. It has gradually been recognized that “too little” or “too much” autophagy activation is detrimental to cells responding to various pathologies [3,4,5]. Achieving a well-balanced and tightly controlled autophagy response requires a deep understanding of its detailed regulatory mechanisms.

The activation of autophagy starts with the formation of an autophagy initiation complex, a critical enzymatic complex mainly consisting of unc-51-like kinase 1 (ULK1), autophagy-related protein 13 (ATG13), and focal adhesion kinase family interacting protein of 200 kD (FIP200) [6]. Early studies effectively documented the regulation of ULK1 and ATG13; however, the essential role of FIP200 in autophagy was not revealed until the late 2000s [7,8], likely due to the lack of a precise equivalent in yeast. FIP200 primarily acts as a scaffold protein, stabilizing the autophagy initiation complex [7,8,9]. It also regulates autophagy through interactions with other mediators for selective autophagy, such as binding to p62 for the degradation of ubiquitinated substrates [10]. However, how FIP200 per se is regulated remains largely unknown.

Using mass spectrometry analysis, we discovered that FIP200 protein was methylated by SET domain containing 2 (SETD2), a methyltransferase, upon autophagy activation. The specific methylation site lysine 1133 (K1133) was exhibited and validated by the specific antibody against K1133. We identified Trim21, an E3 ligase responsible for FIP200 ubiquitination and the subsequent FIP200 protein degradation. The methylation protected FIP200 from Trim21-mediated ubiquitination, stabilizing the protein and promoting autophagy activity. SETD2 knockdown reduced FIP200 methylation, leading to enhanced FIP200 ubiquitination and its degradation and attenuated autophagy activity. The study uncovered a regulatory mechanism to fine-tune autophagy activity through dynamic protein methylation at the autophagy initial complex factor FIP200, providing an additional regulatory layer to ensure fast and precise autophagy flux activity.

## 2. Materials and Methods

### 2.1. Cells, siRNAs, Plasmids, and Antibodies

HEK293 cells (CRL-1573) and HeLa cells (CCL-2) were obtained from ATCC, Manassas, VA, USA. Nontargeting siRNA (D-001810-01-05) and human SETD2 siRNA (L-012448-00-0020) were from GE Dharmacon, Chicago, IL, USA. FIP200 cDNA (ENST00000025008.10) and SETD2 cDNA (ENST00000409792.4) were amplified by PCR from HeLa cDNA and inserted in the p3xFLAG-CMV-7.1 vector. Plasmids with point mutations were constructed using the Q5 site-directed mutagenesis kit (NEB, E0554S, Ipswich, MA, USA). The following antibodies were used: FIP200 (Cell Signaling Technology, #12436, Danvers, MA, USA), GAPDH (Cell Signaling Technology, #5174, Danvers, MA, USA), Flag (Sigma Aldrich, F1804-1MG, St. Louis, MO, USA), SETD2 (Abcam, ab31358, Cambridge, UK), ULK1 (Cell Signaling Technology, #8054, Danvers, MA, USA), ATG13 (Invitrogen, PA5-75682, Waltham, MA, USA), Ub (Santa Cruz Biotechnology, sc-8017, Dallas, TX, USA), LC3 (Cell Signaling Technology, #2775, Danvers, MA, USA), H3K36me3 (Abcam, ab9050, Cambridge, UK), and Trim21 (Cell Signaling Technology, #92043, Danvers, MA, USA). An FIP200 K1133me1-specific polyclonal antibody was produced by ABclonal Technology, Woburn, MA, USA. Three New Zealand rabbits were immunized with the KLH-conjugated peptide antigen C-TLMTIE(K-me1)DQ. Serum was collected, and the antibody was purified by antigen affinity chromatography. The antibody was validated by dot-blot tests against the TLMTIE(K-me1)DQ and TLMTIEKDQ peptides.

### 2.2. Mass Spectrometry

HEK 293 cell lysate was subjected to anti-FIP200 antibody pulldown followed by SDS-PAGE gel separation. The gel was stained with 0.1% Coomassie brilliant blue R250, and gel bands were digested overnight with trypsin following reduction and alkylation with DTT and iodoacetamide. The samples were analyzed by the Ultimate 3000 RSLC-Nano liquid chromatography system from the UT Southwestern Proteomics Core. Raw MS data files were analyzed using Proteome Discoverer v2.4 SP1 (Thermo, Waltham, MA, USA), with peptide identification performed using Sequest HT searching against the mouse protein database from UniProt.

### 2.3. Co-IP Assay

Cells were lysed in NP-40 lysis buffer. Cell lysates were incubated with protein G-Sepharose beads (GE, 17-0618-01, Chicago, IL, USA), IgG, or the indicated antibodies at 4 °C overnight. The beads were washed with NP-40 lysis buffer four times and boiled with LDS sample buffer (Invitrogen, NP00072, Waltham, MA, USA). The samples were then analyzed by Western blot.

### 2.4. Confocal Imaging

HeLa cells with stable expression of GFP-LC3 were seeded on a 35 mm glass-bottom dish (MatTek, P35G-1.5-14-C, Ashland, MA, USA) overnight and then transfected with control siRNA or SETD2 siRNA for 48 h. Cells were treated with 100 nM Bafilomycin A1 for 6 h, washed with cold PBS buffer, and fixed with 4% paraformaldehyde for 30 min at room temperature. The fluorescence was viewed by a Nikon Confocal A1 laser microscope.

### 2.5. Cycloheximide (CHX) Chase Assay

HEK293 cells were transfected with control siRNA or SETD2 siRNA for 36 h. Cells were treated with cycloheximide, a protein synthesis inhibitor, at 20 μg/mL for 0, 6, and 13 h. Cell lysates were analyzed by Western blot.

### 2.6. Ubiquitination Assay

HeLa cells were co-transfected with His-Ub and the indicated plasmids or siRNAs for 48 h. MG-132, a proteasome inhibitor, was used at a final concentration of 10 uM (Sigma Aldrich, M8699) for 12 h. Cell lysates were incubated with protein G-Sepharose beads and the indicated antibodies at 4 °C overnight. The processed samples were analyzed by Western blot.

### 2.7. RT-qPCR

RNA was isolated from HEK 293 cells using a Quick-RNA MiniPrep Kit (Zymo Research, R1055, Irvine, CA, USA). cDNA was synthesized using LunaScript RT SuperMix (NEB, E3010, Ipswich, MA, USA), followed by qPCR using 2x Universal SYBR Green Fast qPCR (Abclonal, RK21203, Woburn, MA, USA). The following primers were used: SETD2-forward CTCCTCCCAAACCAAAAACC, SETD2-reverse GAGTTCCCAGGTCCATCTCA; LC3B-forward GAGAAGCAGCTTCCTGTTCTGG, LC3B-reverse GTGTCCGTTCACCAACAGGAAG; FIP200-forward CAGCACAAAGTTTGGATGAAATGTC, FIP200-reverse CCTGCTGTACTTTCCATCCTTGG; ULK1-forward GCAAGGACTCTTCCTGTGACAC, ULK1-reverse CCACTGCACATCAGGCTGTCTG; ATG13-forward CAGAACTGCTGGTGAGGACACT, ATG13-reverse AGCAGGCTGATAGGAAAGGCGA; GAPDH-forward GAGTCAACGGATTTGGTCGT, GAPDH-reverse TTGATTTTGGAGGGATCTCG.

### 2.8. Quantification and Normalization

The experiments were independently performed three times. Immunoblot bands of the representative results were quantified and normalized to either the input or GAPDH.

### 2.9. Statistics

For two-group comparisons, an unpaired Student’s t-test was used. A value of *p* < 0.05 was considered statistically significant.

## 3. Results

### 3.1. FIP200 Is Methylated at Lysine 1133 upon Autophagy Activation

**Figure 1:**
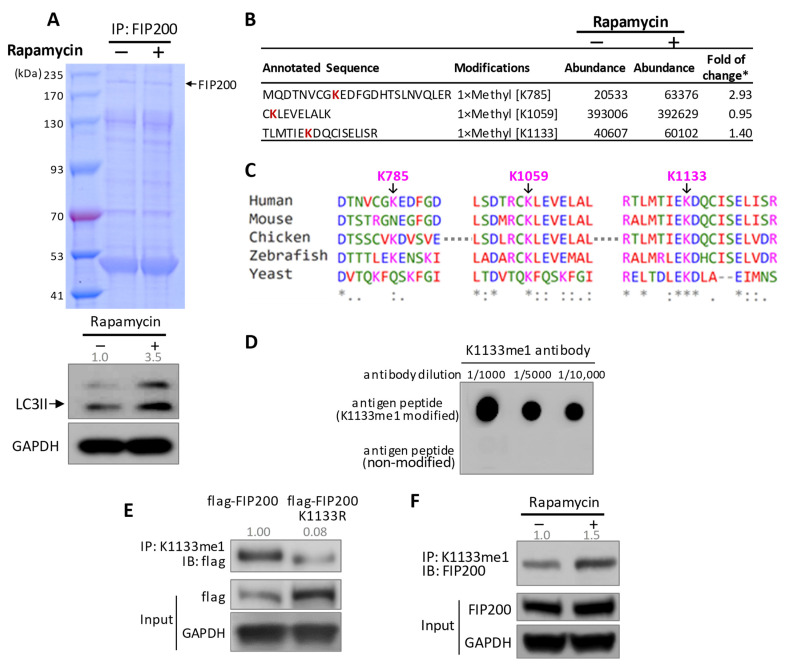
FIP200 is methylated at K1133. (**A**) Pulldown of endogenous FIP200. HEK293 cells were treated with or without 2.5 µM rapamycin for 6 hrs, followed by the pulldown using an anti-FIP200 Ab. Gel bands around 200 kDa were subjected to the PTM-MS (upper panel). Immunoblot showed induced autophagy by Rapamycin (lower panel). LC3II was quantified and normalized to GAPDH. (**B**) PTM-MS analysis identified three FIP200 methylation sites (red-highlighted K). *: fold of change in methylation was normalized by the total FIP200 abundance. (**C**) Evolutionary conservation analysis of the identified methylation sites. *: 100% conserved amino acids among the listed species. (**D**) Dot blot demonstrated a dose-dependent binding of FIP200 K1133me1 Ab to the antigen. K1133me1 Ab was diluted as indicated ratios, and it was only bound to antigen peptide with K1133me1 modification. (**E**) K1133me1 antibody specificity was demonstrated by significantly lower binding to the K1133R mutant than wild-type FIP200. HEK293 cells were transfected with indicated plasmid. The cell lysate was pulled down by anti-K1133me1 Ab and blotted with anti-fIag Ab. Immunoblot was quantified and normalized to the input. (**F**) K1133me1 level was elevated upon autophagy activation. HEK293 cells were treated with or without 2.5 µM rapamycin for 6 hrs. Anti-K1133me1 Ab was used for the pulldown, and anti-FIP200 Ab was used for the immunoblot. Immunoblot was quantified and normalized to the input.

To obtain the profile of post-translational modifications (PTMs) in FIP200 upon autophagy activation, we performed an immunoprecipitation pulldown assay using an anti-FIP200 antibody (Figure 1A), followed by mass spectrometry (MS) analysis (Figure 1B). We compared the changes in the level of PTMs upon autophagy activation by rapamycin, a potent autophagy inducer [11]. Increases in methylation at lysine 785 (K785me1) and lysine 1133 (K1133me1) were revealed by the MS (Figure 1B and Figure S1). We focused on K1133 in this study, as K1133 is highly conserved among species from yeast to humans (Figure 1C). We generated an antibody specific to K1133me1. The immuno-dot blots validated the antibody specificity with a series of antibody dilutions (Figure 1D). The antibody was further confirmed by the immunoblots against the pulldown using the anti-FIP200 antibody in the cell lysates transient transfected with wild-type (flag-FIP200) and mutant FIP200 with lysine replaced by arginine (flag-FIP200 K1133R), respectively (Figure 1E). Lastly, using the K1133 methylation antibody, we successfully detected a noticeable increase in FIP200 methylation levels in the cells treated with rapamycin (Figure 1F), further confirming the methylation antibody specificity and the K1133 methylation response upon autophagy stimulation.

### 3.2. SETD2 Carries out FIP200 K1133 Methylation

**Figure 2:**
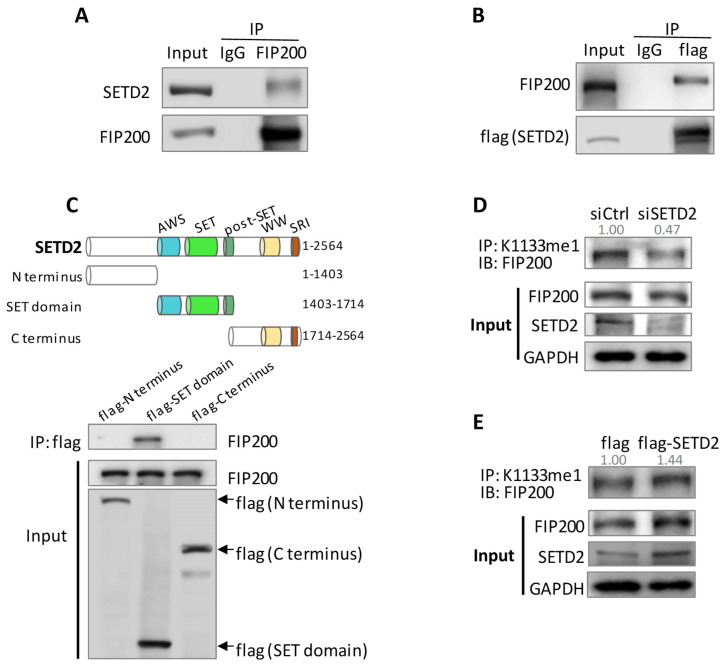
SETD2 binds and methylates FIP200. (**A**) Co-IP assay showed that SETD2 binds to FIP200. HEK 293 cell lysate was pulled down using an anti-FIP200 Ab. SETD2 was detected by immunoblot. (**B**) Co-IP assay showed the interaction between flag-SETD2 and FIP200. Flag-SETD2 was overexpressed in HEK293 cells and pulled down using an anti-flag Ab. FIP200 was detected by immunoblot using anti-FIP200 Ab. (**C**) SETD2 SET domain bound to FIP200. SETD2 protein structure and functional domains were summarized in the schematic graph. Flag-tagged SETD2 fragments were overexpressed in HEK293, pulled down by an anti-flag Ab. FIP200 immunoblot indicated the SET domain, but not the N-terminus and C terminus bound to FIP200. (**D**,**E**) Pulldown assay showed that FIP200 K1133me1 level was positively correlated to the SETD2 level. HEK293 cells were transfected with siSETD2 (**D**) or flag-SETD2 (**E**). Pulldown assay showed decreased and increased FIP200 K1133me1 after SETD2 knockdown and overexpression, respectively. Immunoblot was quantified and normalized to the input.

Our previous study demonstrated that methyltransferase SETD2 was critical for skeletal muscle cell proliferation and differentiation, and the pulldown assay followed by mass spectrometry revealed FIP200 as a possible target for SETD2 (Figure S2) [12]. We performed a set of Co-IP assays to confirm this observation and showed mutual bindings between FIP200 and SETD2 (Figure 2A,B). We dissected SETD2 into three functional fragments: the N terminus, the enzymatic SET domain, and the C terminus. We found that only the SET domain could bind FIP200 (Figure 2C), implying a possible enzyme–substrate relationship between SETD2 and FIP200. We then manipulated SETD2 expression levels by knockdown and overexpression and detected a decline and an increase in K1133 methylation levels, respectively (Figure 2D,E). Together, SETD2 binds to FIP200 and is responsible for FIP200 K1133 methylation.

### 3.3. SETD2-Mediated FIP200 Methylation Maintains FIP200 Protein Stability

**Figure 3:**
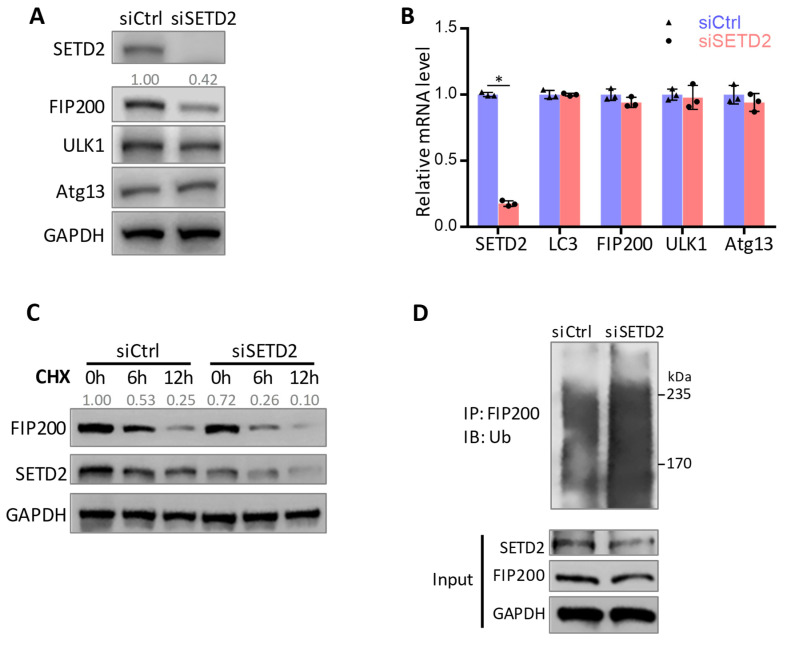
SETD2 maintains FIP200 protein stability. (**A**) SETD2 inactivation resulted in a reduced FIP200 protein level. Expression levels of autophagy initiation complex components were assessed by immunoblot in Hela cells transfected with SETD2 siRNA or scramble siRNA. FIP200 was quantified and normalized to GAPDH. (**B**) RT-qPCR showed that mRNA levels of the autophagy-related genes were not affected by SETD2 knockdown. *: *p* < 0.05. (**C**) FIP200 protein stability was diminished by SETD2 knockdown, shown by Cycloheximide (CHX) chase assay. FIP200 was quantified and normalized to GAPDH. (**D**) FIP200 ubiquitination level was increased upon SETD2 inactivation. HeLa cells were transfected with His-Ub and indicated siRNA.

We next investigated the effects of K1133 methylation on FIP200. We found that the knockdown of SETD2 led to reduced FIP200 protein (Figure 3A) but not a reduction in its mRNA level (Figure 3B). The SETD2 deficiency did not affect the protein and mRNA levels of ULK1 and Atg13, the critical autophagy initiation complex factors, or the LC3 mRNA level (Figure 3A,B), suggesting minimal transcription regulation by SETD2 in these genes. The CHX chase assay was performed to determine the rate of FIP200 protein degradation when protein synthesis was inhibited. We observed accelerated FIP200 degradation when SETD2 was knocked down (Figure 3C). Consistently, an increased FIP200 ubiquitination level in the ubiquitination assay was detected when SETD2 was knocked down (Figure 3D). The data suggest that SETD2-mediated FIP200 methylation protects FIP200 protein from ubiquitination and degradation.

**Figure 4:**
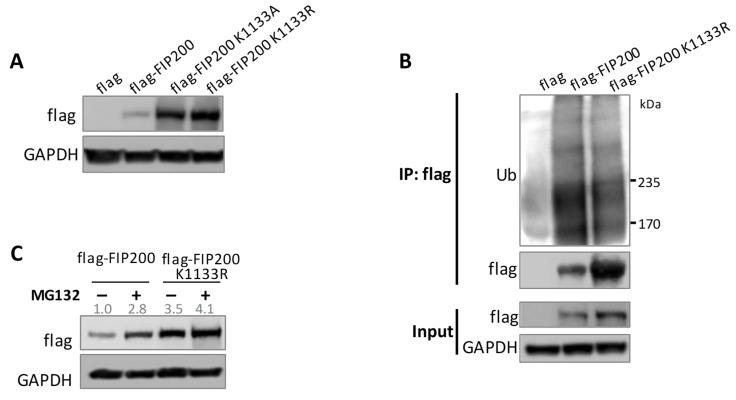
K1133 is critical for FIP200 protein stability. (**A**) Immunoblot of flag-FIP200 and its two mutants showed that K1133 mutations stabilized FIP200. (**B**) The ubiquitination level of the FIP200 K1133R mutant was decreased compared to wild-type FIP200. HeLa cells were transfected with His-Ub and indicated plasmids. (**C**) Immunoblot showed a more significant accumulation of flag-FIP200 compared to the K1133R mutant upon blocking protein degradation. HeLa cells were transfected with indicated plasmids. Flag-FIP200 and K1133R mutant were quantified and normalized to GAPDH.

To further understand the biological significance of K1133 methylation, we generated two FIP200 K1133 mutants with the lysine replaced by alanine (K1133A) and arginine (K1133R), respectively, as arginine is more similar to lysine in terms of charge and structure. The mutations resulted in enhanced FIP200 protein stability with less FIP200 ubiquitination (Figure 4A,B). The result was further confirmed in MG132 treatment when UPS-mediated protein degradation was inhibited by MG132 treatment (Figure 4C). Meanwhile, the data also suggested the possible involvement of FIP200 ubiquitination at K1133.

Together, the data demonstrated that the methylation of K1133 protected FIP200 from UPS-induced protein degradation.

### 3.4. Trim21 Is the E3 Ligase Responsible for FIP200 Ubiquitination

**Figure 5:**
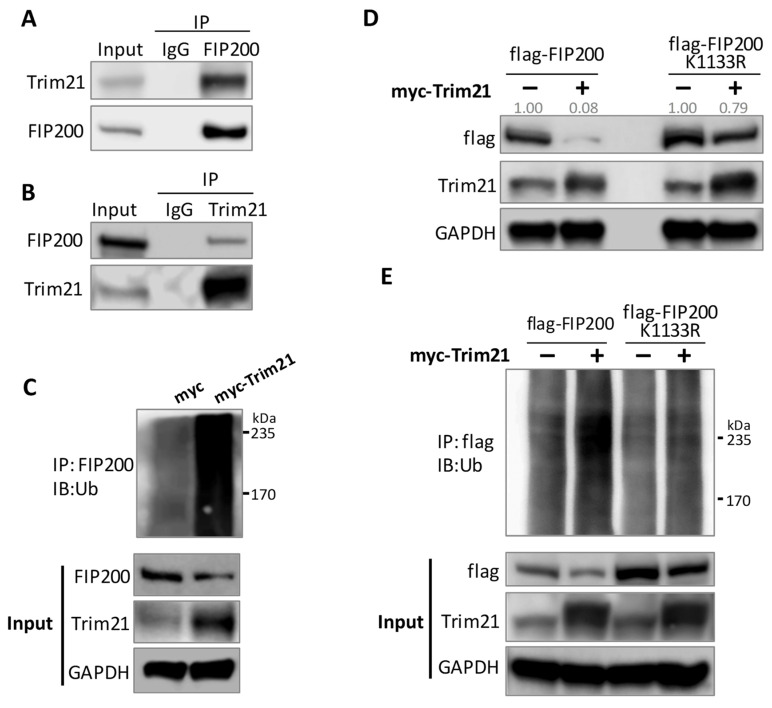
Trim21 ubiquitinates FIP200 for degradation. (**A**,**B**) Co-IP assay demonstrated interaction between endogenous FIP200 and Trim21. Anti-FIP200 Ab (**A**) or anti-Trim21 Ab (**B**) was used for immunoprecipitation. The mutual pulldown was detected by immunoblot. (**C**) Ubiquitination assay indicated Trim21 overexpression increased FIP200 ubiquitination level. HeLa cells were transfected with His-Ub and indicated plasmids. (**D**) Effect of the K1133R mutation on Trim21-induced FIP200 degradation was determined by immunoblot. HeLa cells were transfected with indicated plasmids. Here, a 3-fold amount of flag-FIP200 plasmid was transfected compared to the amount of K1133R mutant. Flag-FIP200 and K1133R mutant were quantified and normalized to GAPDH. (**E**) Ubiquitination assay showed that K1133R mutation reduced FIP200 Trim21-mediated ubiquitination. HeLa cells were transfected with His-Ub and indicated plasmids.

To identify the E3 ligases for FIP200, we performed a pulldown assay in cells transfected with flag-FIP200, followed by an MS analysis of the total proteins bound to FIP200 (Figure S3A). The flag-FIP200 K1133R mutant expression vector was used in the pulldown and MS experiments as an additional control. Among the identified E3 ligases, Trim21 was the most abundant E3 ligase, with the highest coverage percentage, and the mutation K1133R lowered the Trim21 binding affinity to FIP200 (Figure S3B). Trim21’s binding to FIP200 was validated by Co-IP assays (Figure 5A,B). The ubiquitination assay confirmed that Trim21 promoted FIP200 protein ubiquitination and degradation in the cells with a forced expression of Trim21 (Figure 5C,D). Consistent with the pulldown and MS data, the K1133R mutation attenuated Trim21-mediated FIP200 ubiquitination and degradation (Figure 5D,E). The data suggested that the K1133 can be targeted by methylation and ubiquitination.

### 3.5. SETD2 and Trim21 Regulate Autophagy Together through FIP200 Protein Stability

**Figure 6:**
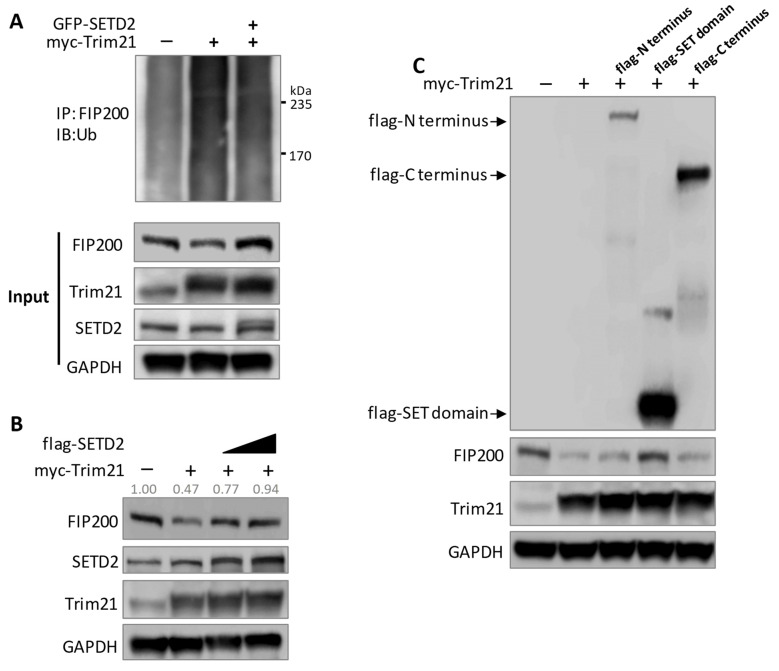
SETD2 reverses Trim21-mediated degradation of FIP200. (**A**) SETD2 overexpression reduced Trim21-mediated FIP200 ubiquitination. HeLa cells were transfected with His-Ub and indicated plasmids. (**B**) SETD2 prevented Trim21-induced FIP200 degradation in a dose-dependent manner. HEK293 cells were transfected with indicated plasmid. Protein levels of FIP200 were assessed by immunoblotting. FIP200 was quantified and normalized to GAPDH. (**C**) The SET domain of SETD2 diminished Trim21-induced FIP200 degradation. HEK293 cells were transfected with indicated plasmid expressing different SETD2 fragments. Immunoblot showed that only the SET domain stabilized the FIP200 protein.

We demonstrated that SETD2 methylates FIP200 to enhance protein stability, while Trim21 ubiquitinates FIP200 to promote its degradation. To determine the relationship between the two regulatory mechanisms involved in FIP200, we forced the expression of SETD2 and Trim21 in the cells. We observed again that the overexpression of Trim21 increased FIP200 ubiquitination and decreased the FIP200 protein level (Figure 6A,B). However, the Trim21-mediated effect was diminished by the overexpression of SETD2 (Figure 6A,B). To further confirm the enzymatic role of SETD2, we transfected cells with plasmids expressing the SETD2 N-terminus, the methyltransferase SET domain (without POLII and histone-3-binding capability), and the C-terminus (containing POLII binding SRI domain). Only the SET domain successfully blocked Trim21-induced FIP200 degradation (Figure 6C), suggesting a minimal epigenetic mechanism mediated by SETD2.

**Figure 7:**
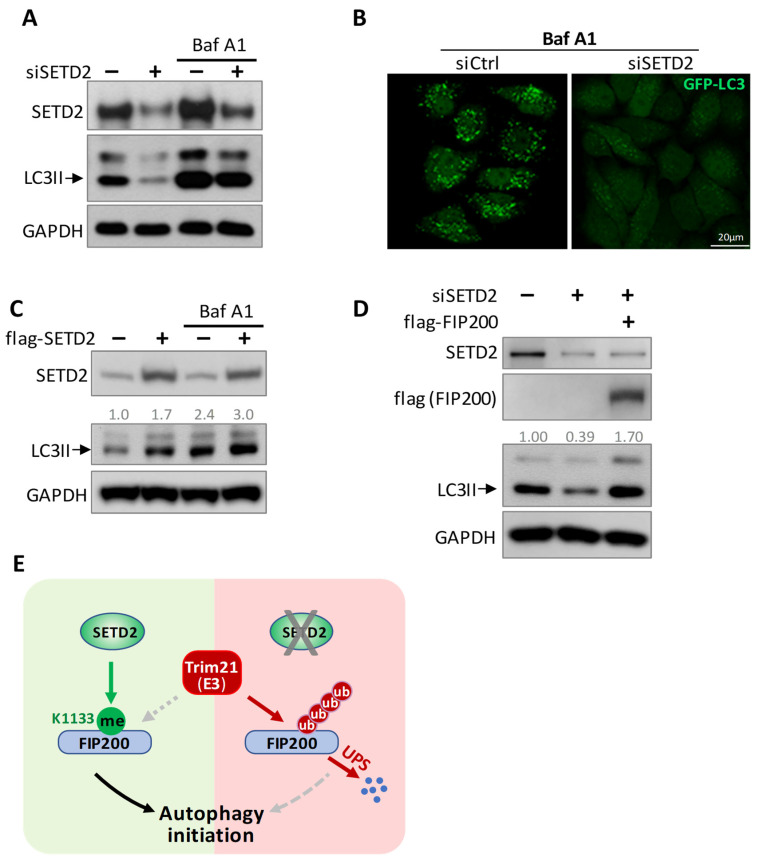
SETD2 promotes autophagy initiation. (**A**) Impaired autophagy initiation after SETD2 knockdown. HEK293 cells were treated with Bafilomycin A1 (Baf A1) to inhibit autophagosome-lysosome fusion. The level of autophagy marker, LC3II, was exhibited by immunoblot. (**B**) Representative images of HeLa GFP-LC3 cells showed diminished autophagosome formation upon SETD2 inactivation. Cells were transfected with indicated siRNAs, and Baf A1 was added to inhibit autophagosome-lysosome fusion. (**C**) SETD2 overexpression promoted autophagy activation. LC3II was quantified and normalized to GAPDH. (**D**) FIP200 overexpression preserved autophagy initiation after SETD2 knockdown. LC3II was quantified and normalized to GAPDH. (**E**) protein Working model. During autophagy initiation, SETD2 methylated FIP200 at K1133, stabilized FIP200, and promoted autophagy initiation. Without SETD2, FIP200 was ubiquitinated by Trim21 and degraded by the ubiquitin-proteasome system, leading to impaired autophagy.

Finally, we examined the biological effects of SETD2-mediated autophagy initiation. The autophagy marker, LC3II, and autophagosome levels were assessed. SETD2 knockdown decreased LC3II and autophagosome levels (Figure 7A,B). To further clarify whether the LC3II reduction was due to compromised autophagy initiation or by enhanced autophagosome degradation, bafilomycin A1 (Baf A1), an autophagosome–lysosome fusion inhibitor, was used, which can prevent LC3II degradation. As expected, the accumulation of LC3 II levels was shown in the control group after Baf A1 treatment. However, we continued to detect the decreased LC3II level in SETD2 knockdown cells compared to the wild-type control cells, even with Baf A1 treatment (Figure 7A,B), indicating compromised autophagy initiation upon SETD2 insufficiency. In contrast, SETD2 overexpression enhanced LC3II levels (Figure 7C). These data confirmed that SETD2 preserves autophagy initiation. In addition, we tested if reintroducing FIP200 into the SETD2-silencing cells could rescue the attenuated autophagy initiation. Our result showed that FIP200 overexpression could preserve the impaired autophagy initiation (Figure 7D), further confirming that SETD2 targets FIP200 to regulate autophagy initiation.

Collectively, these data showed opposite regulations mediated by SETD2 and Trim21 to achieve a balanced autophagy activation (Figure 7D). The mechanism helps maintain cellular homeostasis and efficiently engages in various biological processes.

## 4. Discussion

Unlike the “simple” up- or downregulation of genes at the transcription level, PTMs regulate protein functions and activities from multiple layers. As a result of various PTMs, a protein’s capabilities are maximized, along with more precise and dynamic regulations. The autophagy initiation complex, which mainly consists of ULK1, ATG13, FIP200, and the accessory subunit ATG101, has been shown to undergo multiple PTMs, particularly phosphorylation, during autophagy flux [13,14,15]. PTMs of ULK1 are the most studied because it was not only the first autophagy-related protein discovered but it also functions as a kinase to modify itself and other components in the complex [16,17]. Most PTMs on ULK1 directly activate or inhibit its kinase activity, such as phosphorylation by mTOR and AMPK [18]. Ubiquitin modifications also control ULK1 protein stability. Cul3-KLHL20, MUL1, and NEDD4L ubiquitinate ULK1 for degradation, while TRAF6 catalyzes K-63-linked poly-Ub on ULK1 to maintain its stability [19].

Unlike ULK1, FIP200 is a recently identified essential autophagy-related protein and a component of the ULK-ATG13-FIP200 autophagy initiation complex [7,8]. PTMs on FIP200 are rarely deliberated, while several global phosphorylation site mappings have revealed several suggestive FIP200 phosphorylation sites with no further validation [20,21,22,23]. Current knowledge about FIP200 in autophagy is limited, and it is generally believed that FIP200 mainly functions as a scaffold protein for recruiting other cofactors to the autophagy activation machinery. Considering FIP200 as an “assembler” seems to be the reason to overlook its importance. Recent studies continue to exhibit new regulatory roles of FIP200 in autophagy, including promoting the selective degradation of ubiquitin condensates through the interaction of p62, TAX1BP1, CCPG1, and Rab5 [10,24,25,26]. In this study, we used unbiased mass spectrometry approaches to reveal K1133 methylation at FIP200 and elucidated the biological significance of K1133 methylation. The K1133 site is highly conserved among species from yeast to humans, implicating K1133 methylation as an ancient regulatory mechanism involved in autophagy activation. Interestingly, our mass spectrometry analysis also detected several autophagy-activated phosphorylation sites of FIP200 that were suggested by the global phosphorylation mappings, providing candidates for future investigation.

SETD2 (SET domain containing 2) was initially discovered as a huntingtin-interaction protein [27], and it later demonstrated methyltransferase activity at histone 3 lysine 36 (H3K36) [28]. A SETD2 deficiency or defect causes a variety of pathological conditions, especially tumors [29]. It is generally believed that SETD2 is the only enzyme that specifically trimethylates lysine 36 of histone H3. Besides its histone methylation, four cytosolic (nonhistone) targets have been reported so far [30,31,32,33]. Our discovery of FIP200 methylation by SETD2 adds one more target to the list, expanding its regulatory roles beyond the histone writer function. SETD2 binds and methylates FIP200 at the K1133 site. SETD2 directly controls the K1133 methylation level and the resultant FIP200 level. The methylation of FIP200 protects the protein from ubiquitination. Interestingly, the methylation and ubiquitination share the same site, lysine 1133, and the two PTMs orchestrate FIP200 protein stability, supporting our proposed model (Figure 7E).

Trim21 was discovered to target multiple proteins for degradation in autophagosomes or proteasomes [34,35]. Most of Trim21’s substrates are engaged in innate immunity and interferon signaling, and the degradation of these modulators, in turn, reforms cell-cleaning activities such as autophagy [36,37,38,39]. Consistent with these studies, the Trim21-mediated FIP200 degradation uncovered here further elucidates a dynamic regulatory mechanism mediated by SETD2 and Trim21 in the ULK-ATG13-FIP200 autophagy machinery.

One limitation of the study is that it cannot completely rule out SETD2-mediated transcription regulation, although we have several lines of evidence to suggest that is not the case. By methylating FIP200 K1133, SETD2 stabilized the FIP200 protein without affecting its transcription level and several other critical factors in the autophagy activation machinery (Figure 3B). We also demonstrated that the SET domain alone (without the histone modification function) was also sufficient to block Trim21-induced FIP200 ubiquitination and protein degradation (Figure 6C), strongly supporting the conclusion. However, we believe that the potential for alternative epigenetic regulation by SETD2 remains open for future investigation. Some interesting questions, such as the relationship between mTOR-mediated autophagy inhibition and SETD2-induced FIP200 methylation and how *Setd2* per se is regulated in response to autophagy activation, are worthy of future investigation. It should be noted that, during the preparation of this paper, one study reported that the loss of SETD2 attenuated autophagy indirectly by targeting actin lysine 68 trimethylation in a human renal cancer cell line [40]. This finding is phenotypically consistent with our observations. Our study uncovered a new regulatory mechanism in autophagy initiation complex activation through SETD2-mediated FIP200 methylation coupling with Trim21. The functional balance between SETD2 and Trim21 effectively regulates autophagy initiation and autophagy-related conditions and diseases.

## Data Availability

To request data, contact us by email: jiangchang@tamu.edu.

## Supplementary Materials

The following supporting information can be downloaded at: https://www.mdpi.com/article/10.3390/cells11213333/s1, Figure S1: MS spectrums of K785me1, K1059me1, and K1133me1; Figure S2: FIP200 was detected in SETD2 pulldown HEK293 lysate by MS analysis; Figure S3: MS assay for E3 ligases of FIP200.
