# Supplementary material for: FIP200 Methylation by SETD2 Prevents Trim21-Induced Degradation and Preserves Autophagy Initiation
Source: Cells. 2022 Oct 22;11(21):3333. doi: 10.3390/cells11213333 (PMC9653720; doi:10.3390/cells11213333)
Supplement: Supplementary file 1 [file cells-11-03333-s001.zip › cells-1952292-supplementary.pdf]

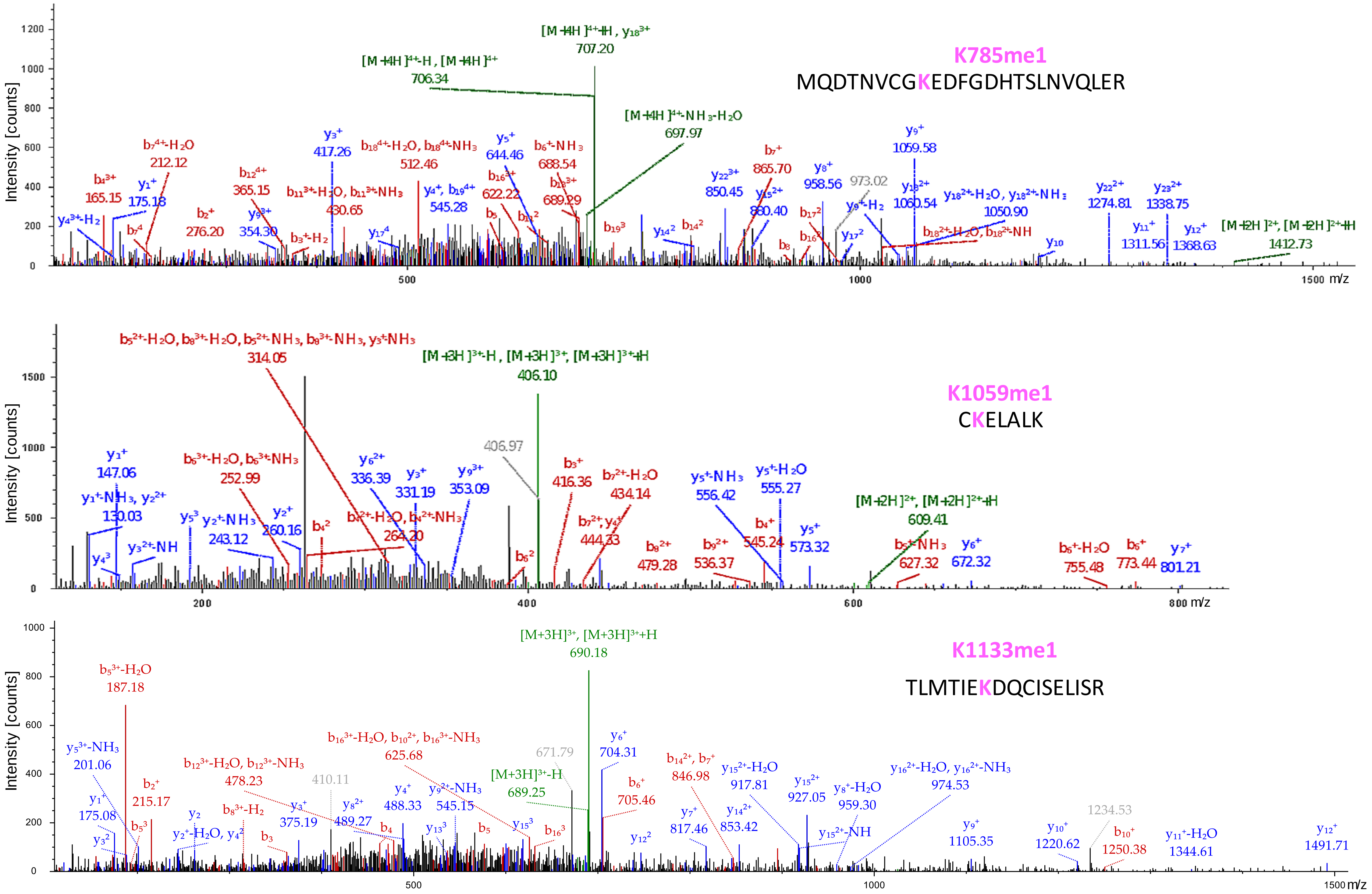

Figure S1. MS spectrums of K785me1, K1059me1, and K1133me1.

Description	Score	Coverag e	# Proteins	# Unique Peptides	# Peptides	# PSMs	MW [kDa]
Histone-lysine N-methyltransferase SETD2 GN=SETD2	1857	19	7	34	38	120	287
FAK Family Kinase-Interacting Protein of 200 kDa GN=FIP200	259	9	3	10	12	14	183

Figure S2. FIP200 was detected in SETD2 pulldown HEK293 lysate by MS analysis.
HEK293 cells were transfected with flag-SETD2 (aa 1148-2564). The cell lysate was pulled down by an anti-flag antibody and then analyzed by MS protein identification. FIP200 was detected in the pulldown lysate.

A

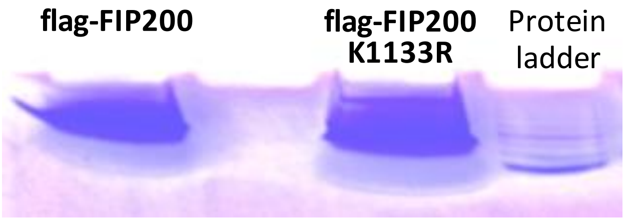

B

Description	Coverage [%]	MW [kDa]	flag-FIP200 Abundance	flag-FIP200 K1133R Abundance	Fold of change
E3 ubiquitin-protein ligase TRIM21	42	54.1	24091754	22079577	0.9
E3 SUMO-protein ligase RanBP2	11	358	9203197	10188113	1.1
E3 ubiquitin-protein ligase RBX1	31	12.3	2158439	2585487	1.2
E3 ubiquitin-protein ligase CHIP	27	34.8	1825182	2987447	1.6
E3 ubiquitin-protein ligase HUWE1	3	481.6	1576028	2105925	1.3
E3 ubiquitin-protein ligase RING1	13	42.4	913517	1013630	1.1
E3 ubiquitin-protein ligase UBR4	2	573.5	910477	793389	0.9
E3 ubiquitin-protein ligase RNF13	6	42.8	760392	814556	1.1
E3 ubiquitin-protein ligase RING2	11	37.6	751760	972852	1.3
E3 ubiquitin-protein ligase RNF220 (Fragment)	3	29.3	660860	643548	1.0

Figure S3. MS assay for E3 ligases of FIP200.

(**A**) HEK293 cells were transfected with flag-tagged wild-type FIP200 or K1133R mutant. Cell lysates were pulled down by an anti-flag antibody, and the pulldown lysate was analyzed by MS.

(**B**) The top ten abundant E3 ligases identified by MS.
